# Application of Queuing Analytic Theory to Decrease Waiting Times in Emergency Department: a review

**DOI:** 10.5812/atr.9859

**Published:** 2013-06-01

**Authors:** Roger Edmund Thomas

**Affiliations:** 1 Department of Family Medicine, Faculty of Medicine, University of Calgary, Alberta, Canada

**Keywords:** Emergency Medicine, Waiting Time, Patients

Dear Editor,

This is a study of a Tehran university hospital emergency department with 50,000 annual visits. The researchers are to be complimented for innovating and the amount of effort invested in the changes (1). A chart review which identified that 47.7% of the patients were referred for trauma treatment, but this finding does not seem to have affected the design of the subsequent study. The first stage involved a two month study of nurses recording demographic items and service times, which were compared to those in the hospital informatics system. No data were presented on the accuracy of the survey compared to the informatics data. The second stage of the study involved recording input times (triage logging, chart recording, and first visit of the resident); throughput (checking the nurses’ orders, requesting and obtaining ECGs, lab tests, ultrasounds, CT scans, X rays and consultations); and output (time of order and time of admission to the ICU, CCU, operating theatre, or transfer to ward, or discharge from the emergency ward). The third stage of the study used ARENA (Version 13.5) software to model the flow of patients through the ED. Five interventions were introduced, but were not implemented in an RCT tried. An additional first year resident was not associated with any change in length of stay, but an additional senior emergency resident on each shift was associated with a decrease in length of stay from 4 to 3.75 hours. An additional clerk to take ECGs in the ED reduced performance time from 26 to 18 minutes. A 50% increase in laboratory staff was associated with a reduction in average stay of 45 minutes. “Increasing consultation capacity” by 50% was associated with a decrease in average ED stay by 45 minutes, and increasing both laboratory staff and consultation capacity with a decrease by 90 minutes. This is a study associated with increases in resources and some dramatic changes in time in the ED. No evidence was presented about the patient numbers affected by each intervention, or the patients’ presenting problems and acuity during the interventions. No evidence was provided that the intervention Protocol was implemented using a manual of procedures, or that the interventions were monitored for fidelity. We do not know the details of patient care during the five interventions, or why the interventions were or were not associated with changes in waiting times or total time in the emergency department. Three important systematic reviews on improvements in patient flows in EDs were published before this article, and are not referenced. Oredsson (2) undertook a systematic review of the literature to 31 March 2009 and identified 33 articles with a total of > 800,000 patients. They assessed internal validity, precision and external validity, and used the GRADEPRO system for overall appraisal. No study fulfilled the criteria for high quality, 22 were of medium and 11 of low quality. For effects on outcome measures they identified one RCT and eight observational studies of interventions to reduce waiting time to see the physician, two RCTs and eight observational studies to reduce total length of stay in the ED, and five observational studies to reduce the number of patients leaving the ED without being seen by a physician. They found moderately strong evidence for each of these groups of interventions. For specific interventions the evidence was positive for fast tracking reducing both waiting time to see the physician and length of stay, but evidence for team triage was limited (except for reducing the number leaving the ED without being seen). Point-of-care laboratory testing reduced laboratory test turnaround time but not length of stay. The evidence for nurses requesting X-rays reducing either waiting time or total length of stay was limited. The systematic review by Harding et al. (3) searched the literature until August 2009, and examined patient flows in several departments. They identified studies of 16 emergency departments, a dental surgery, two sexual health services and an obstetrics unit, and focused on triage interventions. Data quality was assessed using the PEDro scale, and half of the studies were assessed as of poor quality (score < 3/10) and only three RCTs were identified (one randomized the patients and two randomized shifts). They concluded that there was “moderate“ evidence for cases requiring less intensive use of resources that combining triage and initial treatment improved patient flow. A systematic review of any changes associated with the introduction in the UK of a target that 98% of ED patients should be seen within four hours searched the literature 2004-9 and identified eight studies. Six had quantitative data but all were either uncontrolled before-and-after studies or time series. Study quality was assessed using the Newcastle-Ottawa Scale. There was no evidence that introducing the target changed waiting times. However, there was a marked rise (37%) in presentations to emergency departments in the UK National Health Services during the study periods. For the research group at the Tehran university emergency department an excellent goal would be to conduct a thorough review of the literature and systematic reviews in all languages and identify interventions in the literature that correspond to those that are relevant to their ED situation or improve or define more clearly the interventions they tested here. They need to identify all the key features that need to be implemented in a protocol and develop a manual to guide and monitor 100% implementation. They can make an important contribution to the literature by conducting an exemplary randomized control trial. They should randomize patients. An expert in the design and implementation of RCTs should ensure that the techniques advised in the Cochrane Collaboration Handbook (4) are followed. This will then be a major contribution to the care of patients in Tehran and a contribution to the literature.

## References

[1] Alavi-Moghaddam M, Forouzanfar R, Alamdari S, Shahrami A, Kariman H, Amini A (2012). Application of Queuing Analytic Theory to Decrease Waiting Times in Emergency Department: Does it Make Sense?. Arch Trauma Res..

[2] Oredsson S, Jonsson H, Rognes J, Lind L, Goransson KE, Ehrenberg A (2011). A systematic review of triage-related interventions to improve patient flow in emergency departments.. Scand J Trauma Resusc Emerg Med..

[3] Harding KE, Taylor NF, Leggat SG (2011). Do triage systems in healthcare improve patient flow? A systematic review of the literature.. Aust Health Rev..

[4] Higgins JPT, Green S (2011). Cochrane Handbook for Systematic Reviews of Interventions..

